
JOURNAL INFORMATION
==============================
NLM Title Abbreviation: Northwest Med Surg J
NLM Journal ID: 0020727

The North-Western Medical and Surgical Journal

Publisher: Biomedical Journal Digitization Project

ARTICLE INFORMATION
==============================
PMCID: PMC9943110
Article version: 1
Subjects: Editorial and Miscellaneous

Obituary

Print publication date: 1851 Jul
Volume: 4
Issue: 2
First page: 161
Last page: 161
License: This work is free of known copyright restrictions. For more information, please see PMC Back Issue Digitization.
License URL: https://creativecommons.org/publicdomain/mark/1.0/

==============================

### ARTICLE V.

### CHOLERA.

The cholera appears to be prevailing to a limited extent in the region of St. Louis. In that city there have been about fifteen deaths from it reported daily for two weeks. We learn that it has been prevailing at Alton, Quincy, Springfield and some other points in the southern part of Illinois.

As yet it has not made its appearance in Chicago ; some cases, probably of cholera morbus, have excited the fears of the nervous. An agent has been stationed at LaSalle, by the Board of Health, to give notice of the first approach of the disease from below, when, the Mayor informs us, a quarantine will at once be established. If well conducted we shall have strong hope that we may be spared a visitation of the pestilence the present season.

---
